# A new conceptual framework for the musculoskeletal biomechanics and physiology of ray-finned fishes

**DOI:** 10.1242/jeb.243376

**Published:** 2022-03-08

**Authors:** Ariel L. Camp, Elizabeth L. Brainerd

**Affiliations:** 1 Department of Musculoskeletal and Ageing Science, Institute of Life Course and Medical Sciences, University of Liverpool, Liverpool, L7 8TX, UK; 2 Department of Ecology, Evolution and Organismal Biology, Brown University, Providence, RI 02912, USA

**Keywords:** Muscle power, Suction feeding, Swimming, Locomotion, Performance

## Abstract

Suction feeding in ray-finned fishes requires substantial muscle power for fast and forceful prey capture. The axial musculature located immediately behind the head has been long known to contribute some power for suction feeding, but recent XROMM and fluoromicrometry studies found nearly all the axial musculature (over 80%) provides effectively all (90–99%) of the power for high-performance suction feeding. The dominance of axial power suggests a new framework for studying the musculoskeletal biomechanics of fishes: the form and function of axial muscles and bones should be analysed for power production in feeding (or at least as a compromise between swimming and feeding), and cranial muscles and bones should be analysed for their role in transmitting axial power and coordinating buccal expansion. This new framework is already yielding novel insights, as demonstrated in four species for which suction power has now been measured. Interspecific comparisons suggest high suction power can be achieved in different ways: increasing the magnitude of suction pressure or the rate of buccal volume change, or both (as observed in the most powerful of these species). Our framework suggests that mechanical and evolutionary interactions between the head and the body, and between the swimming and feeding roles of axial structures, may be fruitful areas for continued study.

## Abstract (precis)

**Summary:** The recent discovery that some fish use their whole bodies for high-performance suction feeding prompts a reappraisal of cranial and axial biomechanics and overall body shape in ray-finned fishes.

## Introduction

Suction feeding in ray-finned fishes is an amazing behaviour because it is so fast that to the naked eye, prey simply seem to disappear into the predator's maw. Suction is produced by expansion of the oropharyngeal cavity (buccal and opercular cavities), sucking water and prey in through the mouth aperture. Accelerating a mass of water and prey into the mouth requires substantial force, so suction feeding is both fast and forceful, and therefore requires considerable muscle power (Carroll and Wainwright, 2006; Van Wassenbergh et al., 2015). Where does all that muscle power come from?

One might expect feeding to be powered by muscles in the head, but in most fish these cranial muscles are relatively small and may be insufficient to supply the power required for suction feeding. It has been long known that the epaxial and hypaxial body muscles immediately behind the head contribute to suction feeding (e.g. Liem, 1967; Osse, 1969; Tchernavin, 1948), and many empirical and modelling studies have suggested that axial muscles must be contributing substantial power in many suction-feeding fishes (e.g. Carroll and Wainwright, 2006; Gibb and Ferry-Graham, 2005; Oufiero et al., 2012; Van Wassenbergh et al., 2008). However, it is only in the last few years that we have been able to measure suction expansion power directly to determine empirically the relative contributions of head and body muscles to powering suction feeding (Camp et al., 2015; 2018; 2020; Li et al., 2022 preprint).

The key methodological developments enabling these power measurements have been X-ray reconstruction of moving morphology (XROMM) and fluoromicrometry (Brainerd et al., 2010; Camp et al., 2016; Gatesy et al., 2010). Suction expansion power in Watts can be calculated as the product of the rate of volume change of the buccal cavity (d*V*/d*t*) and the absolute magnitude of sub-ambient buccal pressure (Van Wassenbergh et al., 2015):
(1)

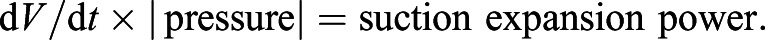


**Fig. 1.**
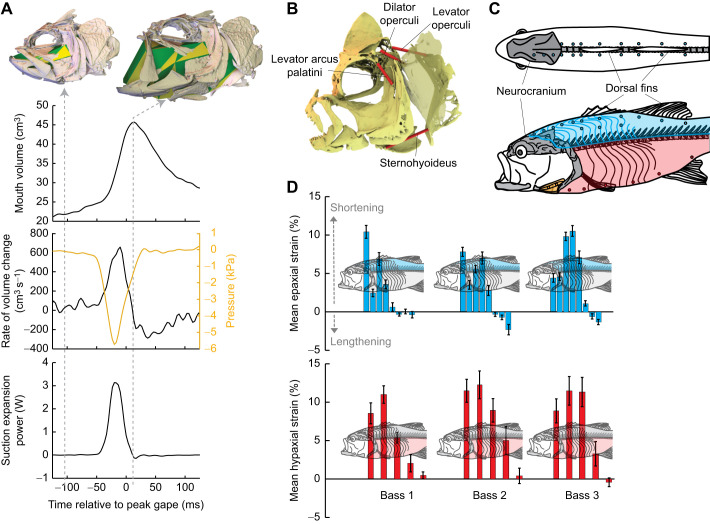
**Measuring suction expansion power and muscle strain.** (A) The dynamic endocast method for measuring instantaneous rate of volume change and suction expansion power, after Camp et al. (2015). (B) Measuring muscle–tendon unit strain with XROMM animations (from Camp et al., 2018). (C) Fluoromicrometry uses intramuscular markers to measure muscle length changes (figure from Camp and Brainerd, 2014). (D) Axial muscle strain during suction feeding in largemouth bass (from Camp and Brainerd, 2014).

Buccal pressure has been measured for many years with liquid-filled cannulae or miniature tip-sensitive pressure transducers (Alexander, 1969a; Lauder, 1980; Norton and Brainerd, 1993). The rate of buccal volume change has been modelled with a range of expanding cone models, with expansion timing determined from external kinematics (Muller and Osse, 1984; Van Wassenbergh et al., 2015, 2006a). With XROMM, we can measure the instantaneous buccal volume change directly from the bones surrounding the buccal cavity (Fig. 1A). Virtual landmarks are placed around the medial surfaces of the bone meshes and the volume of the endocast is calculated on a frame-by-frame basis from the XROMM animation (Camp et al., 2020). This dynamic endocast volume is slightly larger than the actual volume because it does not include the soft tissues and branchial bars that occupy some of the oropharyngeal volume. But it provides a good estimate of the change in volume because the volume of the soft tissues and bones is constant. Thus, XROMM provides empirical measurement of d*V*/d*t*, a key and previously unavailable piece of information for determining suction expansion power.

Prior studies have measured d*V*/d*t* with external kinematics and expanding cone models. One of these studies combined d*V*/d*t* with buccal pressure measurements to calculate instantaneous suction power in largemouth bass, *Micropterus salmoides* (Van Wassenbergh et al., 2015). The magnitudes of predicted power were similar to instantaneous suction power measured with XROMM in largemouth bass (Camp et al., 2015). But in the study based on expanding cones (Van Wassenbergh et al., 2015), pressure peaks well before d*V*/d*t*, whereas in largemouth bass and the three other studied species (bluegill sunfish, channel catfish, royal knifefish), pressure and d*V*/d*t* peak almost simultaneously (Camp et al., 2015, 2018, 2020; Li et al., 2022 preprint). The magnitude of peak power in largemouth bass has also been calculated from geometric estimates of d*V*/d*t* and buccal pressure (Carroll and Wainwright, 2009), yielding similar peak power magnitudes for largemouth bass to those in the studies described above, but much lower magnitudes for bluegill sunfish, *Lepomis macrochirus*: 2–3 W compared with 10–15 W measured empirically (Camp et al., 2018). Suction power has been measured with particle image velocimetry (Avidan et al., 2020 preprint), yielding similar power per body mass results for *L. macrochirus* to those from the XROMM and endocast method (Camp et al., 2018).

For comparing suction expansion power with the available muscle power, it is necessary to know which head muscles and which parts of the epaxial and hypaxial musculature are shortening and generating positive muscle power during peak suction power production (Camp et al., 2015). Muscle strain in the head muscles can be measured directly from the changes in length between the muscle attachment points on the bones (Fig. 1B). This method works when muscles have negligible series elasticity and muscle fibres running directly between the attachment points (Gidmark et al., 2013). For axial musculature, radio-opaque markers implanted in the muscles can be used to measure muscle strain by fluoromicrometry (Fig. 1C; Camp et al., 2016). These segmented muscles extend from the head to the tail, and it is not anatomically obvious which regions are actively shortening during suction feeding. Fluoromicrometry can measure strain at many locations simultaneously to determine the extent of shortening within these muscles.

To date, we have measured suction expansion power and muscle strain in four species: largemouth bass, *M. salmoides*; bluegill sunfish, *L. macrochirus*; channel catfish, *Ictalurus punctatus*; and royal knifefish, *Chitala blanci* (Camp and Brainerd, 2014; Camp et al., 2015; 2018; 2020; Li et al., 2022 preprint). Suction expansion power compared with the maximum potential muscle power production (based on muscle mass) from the primary expansive cranial muscles (sternohyoideus, levator arcus palatini, levator operculi and dilator operculi; we include sternohyoideus here as a ‘cranial’ muscle even though developmentally it is a hypobranchial muscle) shows that the cranial muscles contribute less than 10% and often less than 1% of the power required for the highest power strikes. This means that over 90% of the power must be coming from the axial musculature. Fluoromicrometry has demonstrated that the axial musculature along 60–70% of the length of the body shortens during high-performance strikes (Fig. 1D; Camp and Brainerd, 2014; Camp et al., 2015, 2018, 2020; Li et al., 2022 preprint). For largemouth bass, bluegill sunfish and royal knifefish, both the epaxial and hypaxial muscles contribute to powering suction feeding. The channel catfish does not employ cranial elevation or epaxial muscle shortening for suction feeding. Rather, the epaxial muscles are likely active isometrically to control neurocranium position while the hypaxial musculature shortens to retract the pectoral girdle (Camp et al., 2020). Impressively, channel catfish can still generate suction power similar to that of largemouth bass with just the hypaxial musculature.

Given the tapering body shape of the other three species, with more muscle mass near the shoulder than the tail, axial musculature shortening across the cranial 60–70% of the body means over 80% of the mass of the axial musculature generates power for high-performance suction feeding. The cranial muscles are electrically active and control the skeletal kinematics of buccal expansion, but are too small to contribute substantial power (Camp et al., 2015). Instead, power is transferred from the axial musculoskeletal system to the cranial musculoskeletal system for suction feeding. This power transfer is analogous to athletic feats in many human sports: just as baseball pitchers or cricket bowlers transfer power efficiently from their legs and core to their throwing arms, so fish transfer power from body to head.

## A new conceptual framework for fish musculoskeletal biomechanics and physiology

The discovery that over 80% of the axial muscle mass is generating over 90% of the power for suction feeding, while the cranial muscles are coordinating expansion, suggests a new framework is needed for studying the musculoskeletal biomechanics and physiology of ray-finned fishes. Axial muscles, the pectoral girdle and the vertebral column in fishes have traditionally been studied primarily from the perspective of their role in locomotion. Viewing them as feeding, or at least dual-function, structures offers a novel framework for understanding the biomechanics and physiology of the axial musculoskeletal system. Similarly, viewing the cranial musculoskeletal system as a power transmitter, rather than solely a power generator, may yield new insights into the biomechanics and physiology of cranial systems.

We contend that this framework differs from most current perspectives because the specificity of knowing that nearly the entire mass of axial musculature (over 80%) can contribute effectively all (90–99%) of the power for suction feeding in the most powerful strikes changes the way we should be studying the musculoskeletal biomechanics and muscle physiology of fishes that feed with high-performance suction feeding. Including the axial muscles, pectoral girdle and vertebral column in the feeding system of fishes is a longstanding concept (Liem, 1967; Osse, 1969; Tchernavin, 1948), but most of the work in this area has not been specific about which parts of these systems are engaged in feeding and what they contribute. This may be why most studies to date on fish feeding have focused on the cranial musculoskeletal system (but see exceptions in the next section) and biomechanical studies of fish axial muscles and the vertebral column have been nearly entirely from a locomotor perspective. Insights from our work on suction power suggest that suction feeding studies should not typically stop at the head – that would be like studying only the hand to understand how humans grip a doorknob and turn it. This perspective would miss the contributions of forearm muscles to grip strength and the biceps brachii to supination of the hand. Our results also suggest that axial musculoskeletal form and function in high-performance suction feeders may be just as likely to have been shaped by natural selection for suction feeding as for swimming, and studying the axial musculature and vertebral column as dual-function structures is essential for understanding their physiology, biomechanics and evolution (Camp, 2019; Jimenez and Brainerd, 2020; Jimenez et al., 2021).

The impact of this new framework for studying suction feeding may also extend beyond physiology and biomechanics. Fish trophic morphology and function are used widely as a model system for studying: adaptive radiation (e.g. Clarke and Johnston, 1996; Schluter, 2000; Seehausen, 2006), diversification of complex traits (e.g. Collar et al., 2009; Hulsey et al., 2010), trophic ecology (e.g. Matthews, 2012; Norton et al., 1995; Wootton, 2012), microevolution (e.g. Reznick and Bryga, 1996), macroevolution (e.g. Evans et al., 2021; Longo et al., 2015; Wainwright and Longo, 2017) and the evolution of development (e.g. Hu and Albertson, 2014; Hulsey et al., 2005). The new framework proposed here has the potential to advance these fields by integrating our understanding of cranial and axial structures, overall body form, and the mechanical interactions between swimming and feeding.

## Example applications of the new framework

In the axial and cranial musculoskeletal systems of fishes, this new framework is already changing our perspectives on: (1) the role of the vertebral column in suction feeding; (2) anteroposterior and dorsoventral gradients in axial muscle strain, recruitment and fibre architecture; and (3) the role of the sternohyoideus muscle in suction feeding.

Recent XROMM and video reconstruction of moving morphology (VROMM) studies of suction feeding have shown that the vertebral column in many species bends dorsally in a smooth curve as the neurocranium elevates (Camp, 2021; Jimenez et al., 2018). In largemouth bass and staghorn sculpin, the calculated axis of rotation for the neurocranium relative to a caudal body plane is located well caudal to the craniovertebral joint, suggesting that rotations occur across multiple intervertebral joints (Jimenez et al., 2018). This was confirmed in rainbow trout and Commerson's frogfish, which dorsally rotate the anterior 20–30% (trout) and 60–70% (frogfish) of the intervertebral joints to achieve cranial elevation (Camp, 2021). These are not unexpected results, but discovering some species bend over 50% of the vertebral column in a smooth curve demonstrates this dorsal bending should be viewed as mechanically similar to the lateral bending of the vertebral column during swimming (Jimenez et al., 2021). As noted above, most studies of form and function in fish vertebrae have focused on locomotion (reviewed in Nowroozi and Brainerd, 2014). In contrast, this new perspective from recent studies shows, at least in some species of fishes, the morphology and biomechanics of the cranial regions of the vertebral column should be studied as much for their role in suction feeding as for their role in swimming. We expect there are similar insights to be gained from studying the form, function and evolution of the pectoral girdle in the context of feeding and integration with the skull.

The finding that the vertebral column bends dorsally in a smooth curve during neurocranial elevation also impacts our theoretical and empirical understanding of the anteroposterior and dorsoventral gradients in axial muscle strain, recruitment and fibre architecture (Jimenez et al., 2021). The location of the axis of rotation for neurocranial elevation (Jimenez et al., 2018) and dorsal rotation of the cranial end of the vertebral column (Camp, 2021) together indicate that the full dorsoventral extent of the epaxial musculature may contribute power for suction feeding. Electromyography (EMG) studies have shown that the epaxials are active in both ventral and dorsal regions during the highest performance suction strikes in largemouth bass and bluegill sunfish, with a dorsal to ventral recruitment pattern with increasing feeding performance that is opposite to the ventral to dorsal recruitment found for locomotion (Jimenez and Brainerd, 2020; 2021; McLean et al., 2007). Furthermore, sonomicrometry has demonstrated that the anterior part of the body bends dorsally like a beam during suction feeding (Jimenez et al., 2021), analogous to beam-like lateral body bending during body–caudal fin swimming in fishes (e.g. Shadwick et al., 1999; Wakeling and Johnston, 1999). Beam-like dorsal bending imposes a dorsoventral gradient of longitudinal strain in the epaxial muscle mass that is similar, but orthogonal, to the mediolateral strain gradient generated by lateral bending in locomotion. The mediolateral (i.e. locomotor) strain gradient is known to be counteracted by complex, helical muscle fibre architecture that allows fibres located near the vertebral column to contribute power for fast-start swimming (Alexander, 1969b; Rome et al., 1993; van Leeuwen et al., 2008). The discovery of the dorsoventral strain gradient in feeding suggests that we need to reconsider the fibre architecture and contractile properties of the white axial musculature for its role in suction feeding and not just locomotion (Jimenez et al., 2021).

Studies of the cranial musculoskeletal system have also benefitted from the perspective that in high-performance suction feeding cranial muscles primarily transmit power from the axial muscles and coordinate the way the head expands. For example, the sternohyoideus (SH) muscle has long been viewed as a central contributor to suction feeding, actively shortening to retract and depress the hyoid apparatus (Ferry-Graham and Lauder, 2001; Lauder, 1985). However, the SH remains at constant length or even lengthens slightly during suction expansion in three species of clariid catfishes (Van Wassenbergh et al., 2007) and largemouth bass (Camp and Brainerd, 2014; Carroll, 2004). When the SH is active isometrically or eccentrically, we interpret it as acting like a stiff ligament to transmit power from the axial musculature to the head. By contrast, the SH shortens rapidly and substantially during peak expansion power in bluegill sunfish (Camp et al., 2018), striped surfperch (*Embiotoca lateralis*) (Lomax et al., 2020), channel catfish (Camp et al., 2020) and royal knifefish (Li et al., 2022 preprint). Here, we interpret the SH as both generating power and transmitting power from the axial muscles to the hyoid apparatus (Lomax et al., 2020). For lower power strikes, large and actively shortening SH muscles can provide most or nearly all the power for suction feeding (Camp et al., 2018; 2020; Li et al., 2022 preprint). This power-based perspective is helping us understand how and why SH function varies between species.

## Using power to compare suction feeding function

**Fig. 2.**
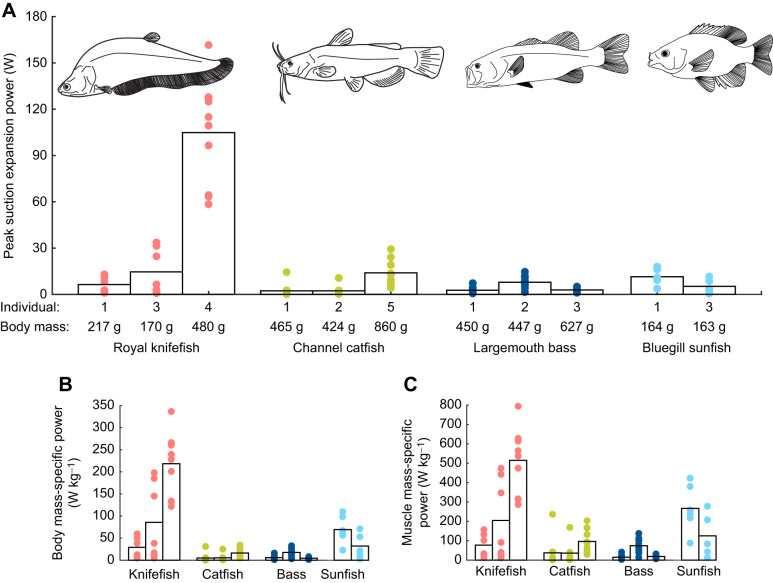
**Comparison of expansion power in four fish species.** (A) Maximum expansion power, (B) maximum power relative to total body mass and (C) maximum power relative to the mass of muscles contributing to suction power (epaxials, hypaxials and sternohyoid for knifefish and sunfish; hypaxials and sternohyoid for catfish; epaxials and hypaxials for bass). All graphs show per-strike values (circles) and average values across all strikes (bars) for each individual of each species: royal knifefish (red, *N*=23 strikes; data from Li et al., 2022 preprint), channel catfish (green *N*=24 strikes; data from Camp et al., 2020), largemouth bass (dark blue, *N*=29 strikes; data from Camp et al., 2015), and bluegill sunfish (light blue, *N*=11 strikes; data from Camp et al., 2018).

The examples above show how the framework is already informing studies of the cranial and axial musculoskeletal systems in individual species. We believe expansion power is also a valuable tool to study suction feeding across species**.** A major challenge in the field is understanding the evolutionary and functional diversity of suction feeding and its relationship with morphology, kinematics and trophic ecology (Holzman et al., 2012; Wainwright et al., 2015). Many valuable perspectives and datasets have already been generated by relating pressure change and mouth expansion during suction feeding to body and mouth morphology (Carroll et al., 2004; Collar and Wainwright, 2006; Higham et al., 2006b; Price et al., 2013; Van Wassenbergh et al., 2006a; Wainwright et al., 2015). Because suction expansion power incorporates both intraoral pressure change and the rate of mouth expansion (Eqn 1), it is another useful tool to link morphology, kinematics and ecology to feeding performance. Suction power data are currently available from four species (Camp et al., 2015, 2018, 2020; Li et al., 2022 preprint). Even this small sample shows substantial variation in maximum recorded suction expansion power among and within species (Fig. 2). Here, we start exploring the possible sources of variation in how these species generate suction power.

First, there is almost certainly a motivational component to maximum recorded power. For example, just within knifefish individual 1, suction power ranges from 60 to 170 W (Fig. 2A). And not all individuals performed to a similar level despite efforts to keep prey type and size, training and appetite constant. The lab-based studies required to measure suction power – with artificial environments and low sample sizes – are unlikely to elicit the true maximal performance for any of these species (Astley et al., 2013). Therefore, we focus on the single individual from each species that generated the highest suction power as our best available estimate of maximal performance.

Second, we expect body size strongly affects suction power. Suction feeding is likely to be power limited (Coughlin and Carroll, 2006; de Jong et al., 1987; Van Wassenbergh et al., 2005) and fish with a larger body mass will have more axial muscle mass to generate power. Therefore, a reasonable null hypothesis is that fish will generate similar amounts of power, relative to their body mass. Yet, there are still large interspecific differences in suction power per unit body mass, with knifefish and sunfish generating much more than bass and catfish (Fig. 2B). This remains true even if accounting for interspecific differences in how much musculature generates power during suction feeding. For example, in catfish the largest body muscle – the epaxialis – does not generate power during suction expansion as in the other three species. Instead of body mass, suction power can be standardized to the total mass of power-producing musculature, but this still shows knifefish and sunfish have more powerful strikes (Fig. 2C). This suggests there are fundamental differences in how these species generate suction power, not simply in the muscle mass available.

**Fig. 3.**
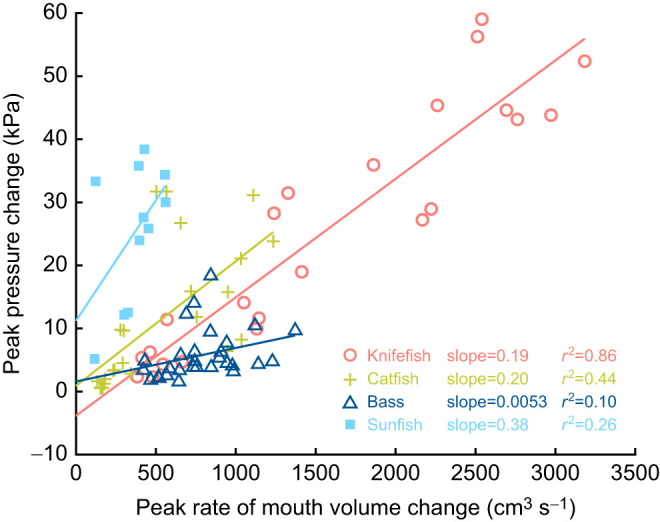
**Peak pressure change as a function of maximum rate of volume change in four fish species.** Positive pressure values represent the peak subambient pressure in the mouth cavity, relative to the initial pressure at the start of the strike. Data are shown for each strike (pooled across individuals) from each species: royal knifefish (red circles, *N*=23 strikes; data from Li et al., 2022 preprint), channel catfish (green crosses, *N*=24 strikes; data from Camp et al., 2020), largemouth bass (dark blue triangles, *N*=29 strikes; data from Camp et al., 2015) and bluegill sunfish (light blue squares, *N*=11 strikes; data from Camp et al., 2018). Linear regression lines (solid lines), slopes and *r*^2^ value were calculated for each species.

One difference is in how these species use pressure change and rate of volume expansion (d*V*/d*t*) in the mouth cavity to generate suction expansion power. High suction expansion power can be produced by a large pressure change, a high d*V*/d*t*, or both (Eqn 1). Sunfish show a wide range of pressures over a small range of d*V*/d*t* values, while bass strikes have the opposite pattern: a wide range of d*V*/d*t* values corresponding to a small range of pressure values (Fig. 3). In contrast, knifefish and catfish vary pressure change and d*V*/d*t* to a similar degree across strikes. The different relationships between pressure change and d*V*/d*t* across species likely reflect their differently sized and shaped mouth cavities (Van Wassenbergh et al., 2006a). Variable pressure and d*V*/d*t* relationships within species such as bass and sunfish likely result from variation in the specific kinematics of how the mouth cavity expands. Even within a single species, the relationships between body size, d*V*/d*t* and pressure can be quite complex (Van Wassenbergh et al., 2006a; 2006b). With power measurements, we can now further investigate these relationships between skeletal kinematics, pressure and d*V*/d*t* and their impact on suction expansion power.

The interspecific variation in body and muscle mass-specific suction power suggests fundamental differences in axial muscle function during feeding. As well as mass, power output also depends on muscle activation, length, velocity and contractile properties. For example, bluegill sunfish not only generated higher mass-specific suction power than largemouth bass (Fig. 2C) but also activate a greater volume of epaxial musculature during feeding (Jimenez and Brainerd, 2020; 2021). Axial muscle architecture and physiology may also be more specialized for powering feeding – rather than swimming – in some fishes. Given the different mechanical demands of suction feeding and lateral body flexion for swimming, it may not be possible to optimize both (Jimenez et al., 2021). Intriguingly, the two species with the highest recorded mass-specific suction power, sunfish and knifefish (Fig. 2C), rely substantially on their paired or median fins for locomotion, rather than body flexion (Gibb et al., 1994; Whitlow et al., 2019). As suction power is measured in more species, future studies can pursue these exciting hypotheses on the relationships between suction power, body shape and axial muscle function.

## Limitations of the suction expansion power framework

Suction expansion power is one way to quantify suction feeding performance, but there are many other valid measures that may be more suitable for some research questions (Holzman et al., 2012). Suction expansion power does not include, for example, flow, prey forces, strike accuracy or body-ram and jaw-ram contributions to suction feeding (Avidan and Holzman, 2021; Holzman et al., 2012; Jacobs and Holzman, 2018; Kane and Higham, 2014; Longo et al., 2015). Furthermore, although we argue here that the expansion power framework is valuable for studying suction feeders, we acknowledge that many species of fishes instead bite, scrape, gouge or otherwise collect food directly with their jaws and teeth.

This framework should not replace other valuable perspectives on suction feeding performance. Performance is far more complex than just power, and depends on the research questions being asked (Holzman et al., 2012). For evolutionary studies, large sample sizes are needed for phylogenetic comparative analyses (Friedman et al., 2020; Longo et al., 2015; Price et al., 2019). Measuring suction power with XROMM is not a high-throughput approach, and other methods such as particle image velocimetry (Jacobs and Holzman, 2018) may provide larger datasets for comparative analyses. Anatomical correlates such as morphological potential or suction index (Carroll et al., 2004; Collar and Wainwright, 2006) are particularly valuable for evolutionary studies because they can be measured on many species. We suggest that the suction power results reviewed here might be used to refine the morphological potential index to include the ventral regions of the epaxial musculature and species that use primarily hypaxial musculature to power suction feeding.

Measures of suction feeding performance that are valuable for ecological and ecomorphological questions are even more diverse. Feeding ecology includes a suite of behaviours and decisions that fish use to locate and acquire food and the behaviour and response of the prey (Holzman et al., 2012). Strike accuracy is a key performance measure (Higham et al., 2006a; Kane and Higham, 2014), as are the timing and coordination of strike kinematics (Holzman et al., 2012). Suction expansion power may add new conceptual perspectives to these measures of ecological performance, but it does not include enough behavioural components to be directly useful for most ecological studies.

## Concluding remarks

We have argued that the specificity of knowing that nearly the entire mass of axial musculature (over 80%) can contribute effectively all (90–99%) of the power for high-power suction feeding offers a new framework for understanding the form and function of suction-feeding fishes. Our framework presents the cranial and axial systems as separate but linked modules. Suction performance will depend on how much power is generated by the axial muscles, and how it is transformed into motion of the cranial skeleton. For example, jaw-ram and suction-based feeding both rely on axial muscle power – the difference is the cranial kinematics that are produced with that power.

Compared with other perspectives on suction-feeding performance, suction expansion power is particularly valuable for comparative biomechanics and physiology studies because it links an output of the system (suction power) to the inputs (muscle power). Muscle power in turn can link to muscle recruitment, muscle physiology and the overall energetics of suction feeding (Carroll and Wainwright, 2009; Jimenez and Brainerd, 2020; Jimenez et al., 2021). Other approaches will continue to be better for ecological and evolutionary studies. But the suction expansion power framework offers a new set of tools for exploring the musculoskeletal biomechanics and physiology of fishes.
